# mTOR pathway mediates the endoplasmic reticulum stress -apoptosis of CD4+ T cell through inhibiting autophagy flux in sepsis

**DOI:** 10.1007/s00011-025-02114-4

**Published:** 2026-02-07

**Authors:** Xianli Lei, Guoyu Zhao, Yawen Xie, Na Cui

**Affiliations:** 1 Department of Critical Care Medicine, Beijing Chao-Yang Hospital, Capital Medical University, Beijing, 100020 China; 2 Department of Critical Care Medicine, State Key Laboratory of Complex Severe and Rare Diseases, Peking Union Medical College Hospital, Chinese Academy of Medical Science and Peking Union Medical College, Beijing, 100730 China

**Keywords:** Sepsis, mTOR, Autophagy, ER-stress, Apoptosis

## Abstract

**Background:**

CD4 + T cells are major reactive subpopulation for cellular and humoral immune responses following sepsis. The apoptosis of CD4 + T cells may contribute to sepsis-induced immunosuppression, and preventing the induction of endoplasmic reticulum stress (ERS) can ameliorate apoptosis of CD4 + T cells in sepsis. The mechanistic target of rapamycin (mTOR) pathway performs an essential regulatory role on ERS-apoptosis of CD4 + T cells. This study aims to elucidate the underlying mechanisms of mTOR regulation of ERS-apoptosis of CD4 + T cells.

**Methods:**

In this study, based on the cecal ligation and puncture (CLP) model, 4-phenylbutyric acid (4-PBA), we firstly detected the percentage of ERS-apoptosis of CD4 + T cells with flow cytometry, Western blotting. Next, we observed the autophagy process and related makers with transmission electron microscopy (TEM) and Western blotting. Furthermore, we created CLP models with T cell-specific mTOR and TSC1 genetic knockout mice, and bafilomycin A1(Baf-A1), a selective inhibitor of autophagy to explore the regulatory role and underlying mechanism of mTOR on ERS-apoptosis of CD4 + T cells. With rapamycin, we proved the clinical potential of mTOR.

**Results:**

Here we observed a considerably higher percentage of apoptotic CD4 + T cells in sepsis, and 4-PBA (an inhibitor of ERS) could alleviate not only ERS, but also the apoptosis of CD4 + T cells. As our previous work proved, deletion of mTOR decreased ERS-apoptosis of CD4 + T cells in sepsis. Furthermore, deficient autophagy, especially impaired autophagic flux was observed in sepsis. Mechanistically, we found knockdown of mTOR erased impaired autophagic flux, decreased ER stress-induced apoptosis, which could be reversed by Baf-A1. More importantly, rapamycin (inhibitor of mTOR) showed great clinical potential.

**Conclusion:**

we proved that mTOR deletion could alleviate CD4 + T cells ERS-apoptosis by rescuing autophagy involving autophagosome –lysosome fusion. For the first time, we demonstrate the mTOR-autophagy-ERS-apoptosis axis in sepsis, enriching the targets for future discovery of new sepsis therapies.

**Supplementary Information:**

The online version contains supplementary material available at 10.1007/s00011-025-02114-4.

## Introduction

Sepsis defined as life-threatening organ dysfunction caused by a dysregulated host response to infection, remains a formidable, worldwide public-health challenge. According to the Global Burden of Disease Study, there were 48.9 million cases of sepsis and 11 million sepsis-related deaths in 2017 [1]. Sepsis treatment still hinges on source control, antibiotics, and multi-organ support therapies (MOST), such as continuous blood purification (CBP) and extracorporeal membrane oxygenation (ECMO), yet mortality is static. This persistent mortality highlights the need to move beyond purely ‘supportive’ care and target the molecular and immunological engines that drive disease progression. A central engine is the rapid oscillation between hyper-inflammation and immunoparalysis. The latter phase is characterized by enhanced cell death, functional exhaustion, and numerical depletion of innate and adaptive immune cells [2]. Among these, CD4⁺ T cells are particularly vulnerable; their loss directly undermines antigen-specific immunity and correlates with late infections and mortality. Consequently, dissecting the mechanisms that precipitate CD4⁺ T cells death—especially apoptosis—has become a priority for restoring immune competence after sepsis [3–5].

One increasingly recognized trigger of lymphocyte apoptosis is endoplasmic-reticulum stress. Under the composite insults of oxidative burst, cytokine storm, and metabolic dyshomeostasis that define sepsis, the ER folding capacity is overwhelmed, misfolded proteins accumulate, and the unfolded-protein response (UPR) shifts from an adaptive program to a terminal, pro-apoptotic cascade [6]. This situation is characterized by three phases: adaptive phase, alarm phase, and finally cell death activation phase [7]. When the stimulus exceeds the cell’s regulatory capacity, it enters the third phase, where RES-induced apoptosis occurs and eventually cell death [8]. Autophagy, a catabolic pathway that continuously removes damaged organelles and aggregated proteins, normally restrains this transition by clearing ER-derived cargo and blunting UPR signaling. Whether autophagy can be therapeutically leveraged to interrupt ERS-apoptosis in CD4⁺ T cells during sepsis is, however, unknown [9]. Based on the current exploration of autophagy and endoplasmic reticulum stress, we would like to further expand the upstream regulatory pathways with the aim of finding key targets that can be interfered with.

The serine/threonine kinase mechanistic target of rapamycin (mTOR) sits at the cross-roads of these two pathways. mTOR is highly regarded for its all-round effects on cell growth, differentiation, metabolism, and autophagy [10]. mTOR synergistically regulates ERS-apoptosis and autophagy pathways in osteoarthritic temporomandibular joint articular chondrocytes [11] and hepatocyte injury induced by TNF-α [12]. We previously showed that genetic deletion of mTOR in CD4⁺ T cells reactivates autophagic flux and improves mitochondrial fitness, whereas loss of its upstream negative regulator TSC1 produces the opposite phenotype [13, 14]. These observations led us to ask whether mTOR activity dictates the balance between ER-stress–induced apoptosis and autophagic cytoprotection in sepsis.

Therefore, we hypothesized that mTOR regulates the onset of ERS-apoptosis through autophagy in sepsis. To verify this hypothesis, we used T cell-specific knockout mTOR and TSC1 (an upstream inhibitor mTOR) mice to create mTOR deletion or overexpression septic models with classical CLP method. Initially, we examined the levels of ERS-apoptosis and autophagy in CD4 + T cells during sepsis. Subsequently, we assessed how mTOR influences them. Further investigation revealed that autophagy flux is involved in the mechanism by which mTOR deletion mitigates ER stress-induced apoptosis. Collectively, our data identify mTOR as a proximal rheostat that links metabolic sensing to ER-stress apoptosis in CD4⁺ T cells during sepsis. By restraining mTOR and thereby re-engaging autophagy, it may be possible to curb lymphocyte attrition, accelerate immune recovery, and ultimately improve survival—offering a mechanistically grounded, translationally tractable adjunct to existing sepsis therapy.

## Materials and method

### Model and experimental process

With male C57BL/6 N mice aged 6–8 weeks and weighing 18–20 g (purchased from Institute of Analysis and Testing, Beijing Academy of Science and Technology (Beijing, China), we created sepsis models, T cell-specific mTOR and TSC1 genetic knockout mice as previous described [15, 16], and performed on account of the National Institutes of Health (Bethesda, MD) guidelines for experimental animals. The classical cecal ligation and puncture (CLP) sepsis models was made following anesthesia, laparotomy(1-2 cm), cecum ligation, puncture (with 20-gauge needle), Cecum contents extrusion, and fluid resuscitation. The sham mice were treated in the same manner as CLP mice, but without cecum ligation and puncture. The mice were randomly divided into related groups, mTOR KO mice were used in the mTOR KO + CLP, mTOR KO + CLP + Baf group (n = 6 in each group). The TSC1 KO mice were used in the Lck-TSC1 + CLP, TSC1 KO + CLP + 4-PBA group (n = 6 in each group). Thirty mice were allocated to each group WT + sham, WT + CLP, CLP + 4-PBA, CLP + Baf, and CLP + Rap group. Mice of the 4-PBA (ERS blocker) group were intraperitoneally injected with 40 mg/kg 4-PBA (T1535CAS1716-12–7; Topscience) 1 h before CLP [17]. Mice of bafilomycin A1(a specific A-L fusion inhibitor) group (1 mg/kg; Solarbio, Beijing, China) were given 1 h following the CLP procedure [18]. Mice of Rapamycin (6 mg/kg; Shanghai yuanye Bio-Technology, Shanghai,China) was administered intraperitoneally 3 h after the CLP operation [14].

### Splenic lymphocyte isolation

12 h after the experimental surgery, the mice were euthanized and the spleens were collected, lymphocytes were isolated according to the instructions of the mouse splenic lymphocyte isolation kit (P8860; Solarbio). The splenocyte suspension was incubated with CD4 + antibody (CD4 + T cells isolation Kit, mouse Miltenyi, 130–104-454), washed, and passed through a separation column for negative screening, and finally centrifuged to obtain CD4 + T cells. The total number of CD4 + T cells was quantified by trypan blue exclusion with a TC20 automated cell counter (Bio Rad). And cell sorting purity was assessed by FACS.

### Transmission electron microscopy (TEM)

The samples were washed three times with 0.1 M phosphate buffer, then fixed with 1% osmium acid at 4℃ for 3 h; then washed three times with buffer, dehydrated step by step with ethanol, replaced with propylene oxide, and embedded with Spurr resin; finally, the samples were polymerized in the oven at 70℃. The embedded blocks were placed on an ultrathin sectioning machine (Leica EM UC6 Ultra-thin Slicer, Germany) for sectioning, the thickness of ultrathin sections was 70 nm, stained with dibasic uranyl acetate and lead citrate, and observed and photographed under a transmission electron microscope (JEM1230, Japan).

### Western blotting

Total proteins from CD4 + T cells were extracted and the concentration was determined by enzyme labeling according to instruction procedures. The equal amounts of total proteins (30 μg) were separated with SDS‐PAGE that then transferred to PVDF (polyvinylidene fluoride) membranes (Millipore, IPVH00010, CA). Then, the samples were blocked with 5% skimmed milk for 1 h and incubated over‐night with 1-2 ml appropriate primary antibodies at 4 °C. After incubation with matched secondary antibody (Goat Anti-Rabbit IgG H&L (HRP) (ab205718) at room temperature for 45 min, the target protein signal was measured using enhanced chemiluminescence (ECL) and analyzed with an image software (Quantity-One, BIO-RAD, U S). The specific protein band expression was quantified and normalized to GAPDH. And the antibodies related in this work will be offered if necessary.

### Apoptosis assay

By staining with FITC Annexin V Apoptosis Detectio (BD) and propidium iodide (PI) (BD), we detected the apoptosis of CD4 + T cells. Stained cells were examined by FACSCalibur (BD) and Cell Quest Pro software. FlowJo software was used to analyze the sample and illustrate the result as a dot plot.

### Statistical analysis

All research data were expressed as mean ± standard deviations (SDs). One-way analysis of variance or two-tailed t-tests was used, followed by Tukey multiple-comparison test was used to analyze multiple groups with GraphPad Prism (V.9.0, GraphPad Prism). When *P* < 0.05 between two group, statistically significant was considered.

## Result

### Significant apoptosis was detected in CD4 + T cells and mediated by ER stress in sepsis

**Fig. 1:**
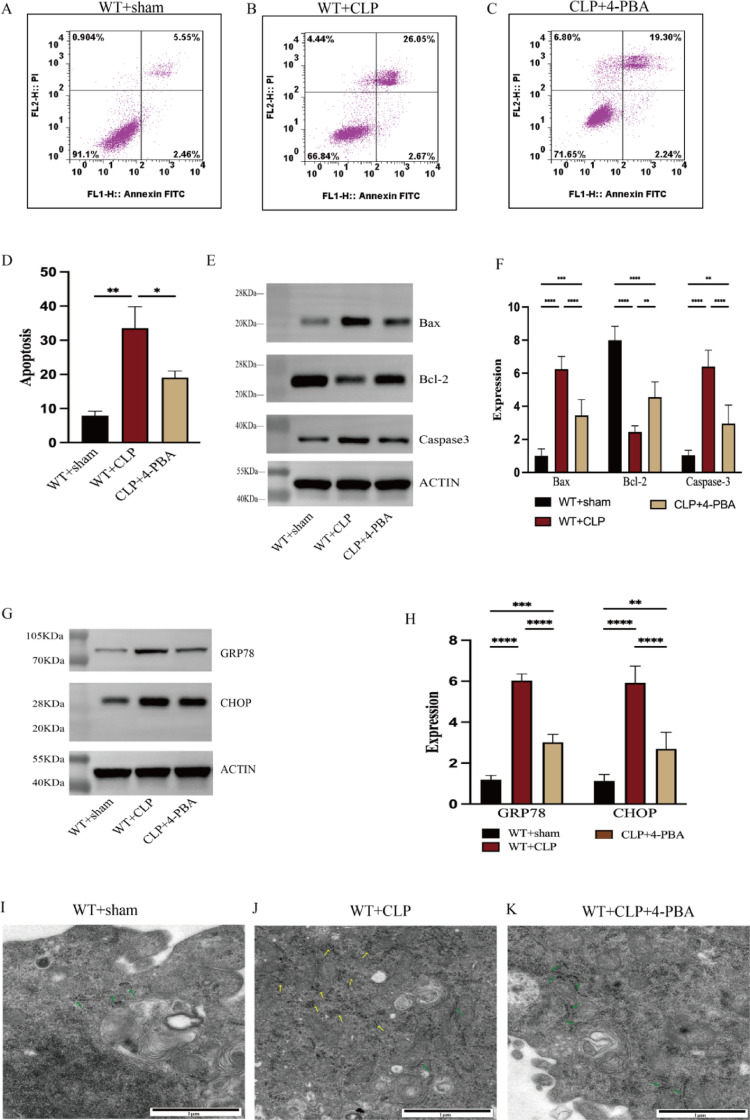
CD4 + T cells apoptosis is increased and correlated with ER stress. **A**–**D** The rate of apoptosis of CD4 + T cells was measured by the ratio of Annexin V-positive and PI-positive/negative CD4 + T cells. **E**–**F** The expression level of bax, bcl2, and caspase 3 were examined by Western blotting. **G**–**H** The expression level of GRP78and CHOP, the marker of ER stress was measured by western blotting. **I**–**K** The microstructure images of ER of CD4 + T cells in WT, WT + CLP, and CLP + 4-PBA mice were observed with electron microscopy. Green arrows represent normal-sized ER. Yellow arrows represent dilation and vesiculation of the ER. Densitometric quantification for expression of protein was normalized to ACTIN protein level. Means ± standard deviations (SDs) of four mice per group are shown. It was deemed statistically significant when *P* < 0.05. *P < 0.05.*P < 0.05, **P < 0.01, ***P < 0.001, ****P < 0.0001

Using flow cytometry with double staining of Annexin V-FITC and PI, and Western blotting, we characterized the apoptosis level of CD4 + T cells and determined the expression of apoptosis-related markers, providing insights into the underlying mechanisms of sepsis-induced immune dysfunction. As compared to the WT group, the CLP group had a considerably higher percentage of apoptotic CD4 + T cells (Fig. 1A, B, D). Furthermore, bax and caspase-3 expression levels were found to be up-regulated in the CLP group, while BCL-2 expression levels were down-regulated, as demonstrated by Western blotting (Fig. 1E, F).

The onset and level of ERS was indicated by the expression of GRP78, CHOP, which were apparently up-regulated in CLP group and which could be down-regulated by 4-phenylbutyric acid (4-PBA) (Fig. 1G, H). Furthermore, as demonstrated by electron microscopy, the CLP group had clear ER stress symptoms, including blistering and expansion of ER structures, which were mitigated by 4-PBA pretreatment (Fig. 1I–K. To verify whether ER stress contributes to apoptosis as described above, mice were treated 4-PBA before undergoing the CLP surgery. The findings indicated that pretreatment with 4-PBA markedly reduced the apoptotic percentage (Fig. 1B–D), and down-regulated the expression of bax and caspase-3 (Fig. 1E, F). Altogether, these results indicate that sepsis was accompanied by elevated ERS and a concurrent increase in CD4⁺ T-cell apoptosis. Notably, inhibiting ERS can effectively mitigate CD4 + T cells apoptosis, highlighting the role of ERS-apoptosis in depletion of CD4 + T cell in sepsis.

### The role of mTOR in ER stress-induced CD4 + T cells apoptosis

**Fig. 2:**
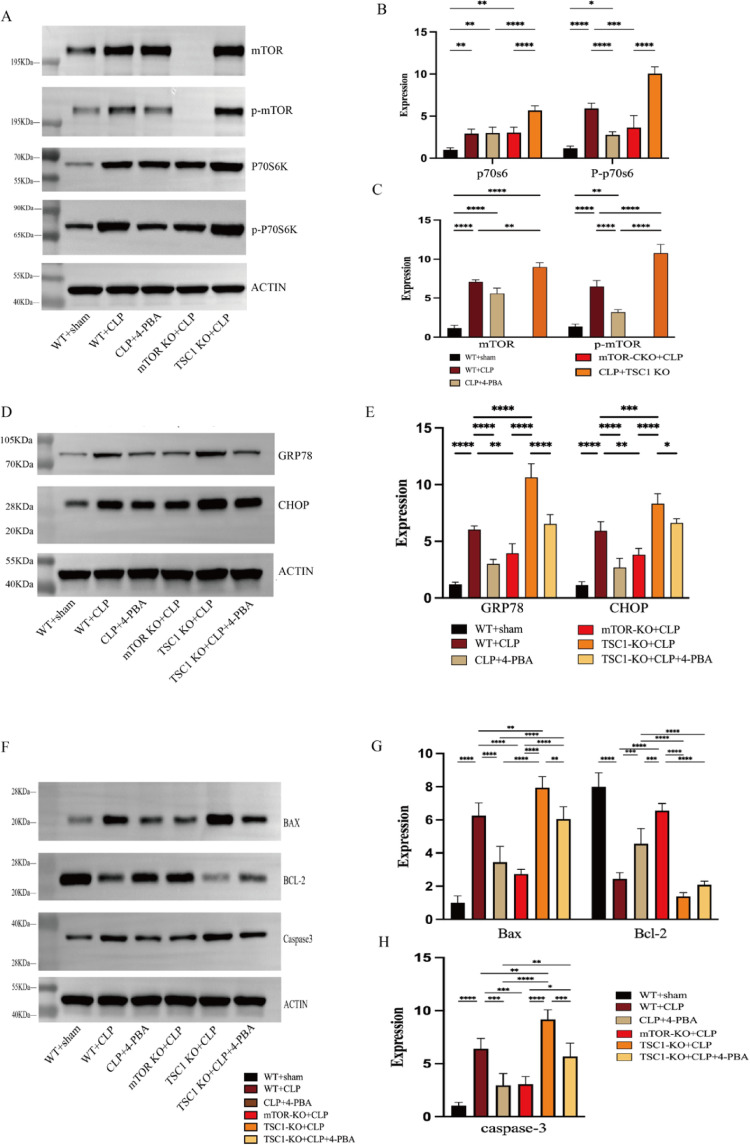
The role of mTOR in ER stress-induced CD4 + T cells apoptosis. **A**–**C** Proteins of mTOR pathway, including mTOR, *P*-mTOR, downstream effectors p70s6k, p-p70s6k were examined by Western blotting. **D**–**E** The expression level of GRP78and CHOP, the marker of ER stress was measured by western blotting. **F**–**H** The expression level of bax, bcl2, and caspase 3 were examined by Western blotting. Means ± standard deviations (SDs) of four mice per group are shown. It was deemed statistically significant when *P* < 0.05. *P < 0.05, **P < 0.01, ***P < 0.001, ****P < 0.0001

**Fig. 3:**
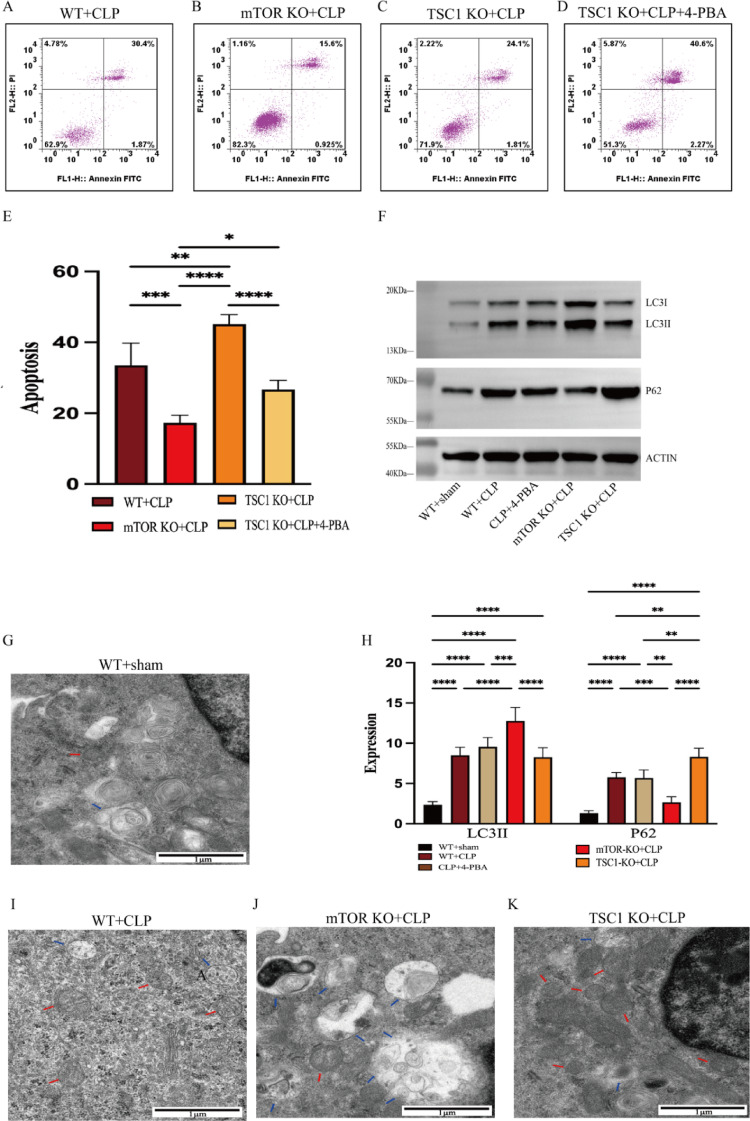
Deficient autophagy is observed under ER stress in sepsis and the role of mTOR on it. (A-E) With flow cytometry, the rates apoptosis of CD4 + T cells were detected in WT + CLP, mTOR KO + CLP, TSC1 KO + CLP, TSC1 KO + CLP + 4-PBA. (F, H). The expression level of LC3I/LC3II and P62, the markers of autophagy process were measured by western blotting. **G**, **I**–**K** Ultrastructural features of CD4 + T cells were investigated using transmission electron microscopy (TEM). In WT group, CD4 + T cells had normal morphologies, revealing baseline autophagy status. WT + CLP mice displayed increased autophagic vacuolization but no significant increase in autolysosome frequency. Large autolysosomes containing abundant contents were seen. More autophagic vacuolization and more autolysosomes were showed in mTOR KO + CLP. Autophagosomes and autolysosomes were fewer in TSC1 KO + CLP mice. Autophagosomes were double-membrane vacuoles containing cytosol or organelles (red arrow). Autolysosomes were single-membrane structures containing digested cytoplasmic components (blue arrow). Means ± standard deviations (SDs) of four mice per group are shown. It was deemed statistically significant when *P* < 0.05. **P < 0.01, ***P < 0.001, ****P < 0.0001

The modulatory role of the mTOR signaling pathway in sepsis has been much argued in previous studies [19]. In the present study, we found that mTOR signaling pathway activity was significantly enhanced (intimated by elevated phosphorylated p70 S6K(or markedly elevated the p-p70 S6K/p70 S6K ratio) and phosphorylated mTOR) in the CLP group compared to the WT group (Fig. 2A–C). In accordance with earlier protocols [13], we created a T cell-specific mTOR/TSC1 knockout mice model which is confirmed by western blotting (Fig. 2A, B), to investigate the function of the mTOR pathway in the control of ER stress-induced apoptosis. In comparison to the CLP and TSC1 knockdown groups, we observed that mTOR knockdown decreased the expression of GRP78 and CHOP (Fig. 2D, E). In contrast to CLP mice, mTOR KO + CLP group showed decreased CD4 + T cell apoptosis rate, and the expression levels of Bax, caspase-3 were downregulated, and the expression level of Bcl-2 increased (Fig. 2F–H), while TSC1 KO group showed enhanced apoptosis rate, and the promotion of apoptosis by mTOR could be blocked by the ER stress inhibitor 4-PBA (Fig. 3A–E). These findings imply the inhibition of mTOR pathway inhibition decreased ERS-apoptosis of CD4 + T cells in septic mice.

### Significant impairment of autophagy is noted in sepsis

Previous studies have shown that endoplasmic reticulum stress can launch autophagy to remove accumulated proteins and defend cells from damage or even death [20]. We observed the protein level of P62, LC3-II and LC3-I, which are markers for autophagy. If both P62 and LC3-II exhibit simultaneous elevation, it implies the inhibition of autophagic flux [21]. In CLP group, sepsis was observed to increase both the LC3-II/LC3-I ratio and the expression of p62, indicating decreased autophagic flux (Fig. 3F–H). In the meantime, we used transmission electron microscopy (TEM) to study the ultrastructure linked to the onset of autophagy and discovered that the CLP group exhibited an accumulation of double-membrane structures which was also a symptom of poor autophagic flux (Fig. 3G, I–K). The above results suggest an autophagy disorder in sepsis. Given mTOR’s role in regulating autophagy, we next asked whether autophagy impairment was involved in the effect of mTOR on ERS-apoptosis.

### mTOR deletion restores autophagic flux to alleviate ERS-apoptosis

**Fig. 4:**
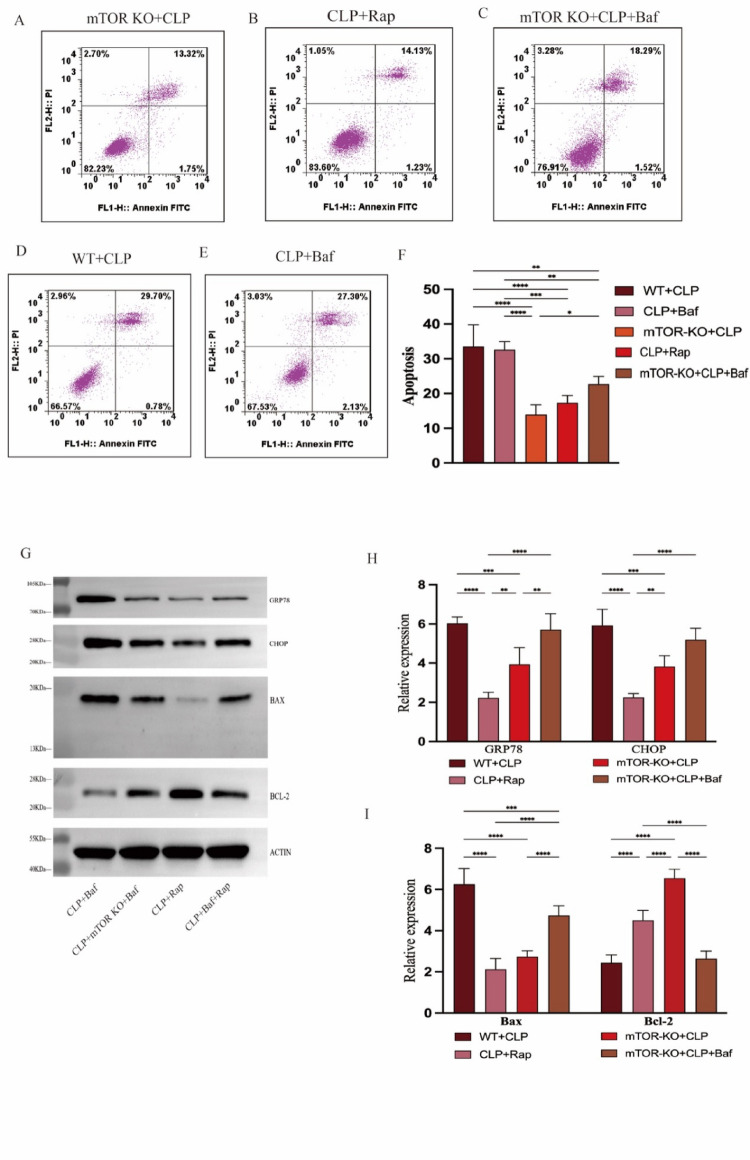
mTOR deletion activates autophagy to alleviate ER stress-induced apoptosis. (A-F) The proportion of apoptosis of CD4 + T cells were detected in WT + CLP, mTOR KO + CLP, CLP + Rap, mTOR KO + CLP + Baf, CLP + Baf by flow cytometry analysis. Densitometric quantification for expression of protein was normalized to ACTIN protein level. **G**–**I** The expression level of GRP78, CHOP, bax, and bcl2, the marker of ER stress and apoptosis were measured by western blotting. Data was presented as means ± standard deviations (SDs) of four mice per group are shown. It was deemed statistically significant when *P* < 0.05. **P < 0.01, ***P < 0.001, ****P < 0.0001

When comparing group mTOR KO + CLP to CLP mice, LC3 II/I expression was considerably higher and p62/SQSTM1 expression was reduced in CD4 + T cells. This indicates that mTOR deletion alleviated the autophagic flux abnormality that was caused during sepsis (Fig. 3F, H). We have shown that the mTOR pathway regulates ERS-apoptosis. To further evaluate whether mTOR deletion alleviates ERS-apoptosis through inducing autophagy, we employed bafilomycin A1, a selective inhibitor of autophagosomes-lysosomes(A-L) fusion. When bafilomycin A1 was administered to mTOR KO + CLP mice, the apoptotic rate of CD4 + T cells was higher than in mice that did not receive bafilomycin A1 (Fig. 4A, F) and the expression of bax and bcl2 illustrates the same results (Fig. 4G, I). As the results showed, pretreatment with bafilomycin A1 increased the expression of GRP78 and CHOP indicating the enhancement of ERS compared with mice in mTOR KO + CLP group (Fig. 4G, H). The above outcome showed that bafilomycin A1 could offset the protective effects of mTOR deletion against ERS-apoptosis of CD4 + T cells in sepsis, and we can say that the protective effects of mTOR deletion on ER stress-induced apoptosis is partly through regulation of autophagy flux.

### Targeting mTOR improves prognosis and shows great clinical therapeutic potential

**Fig. 5:**
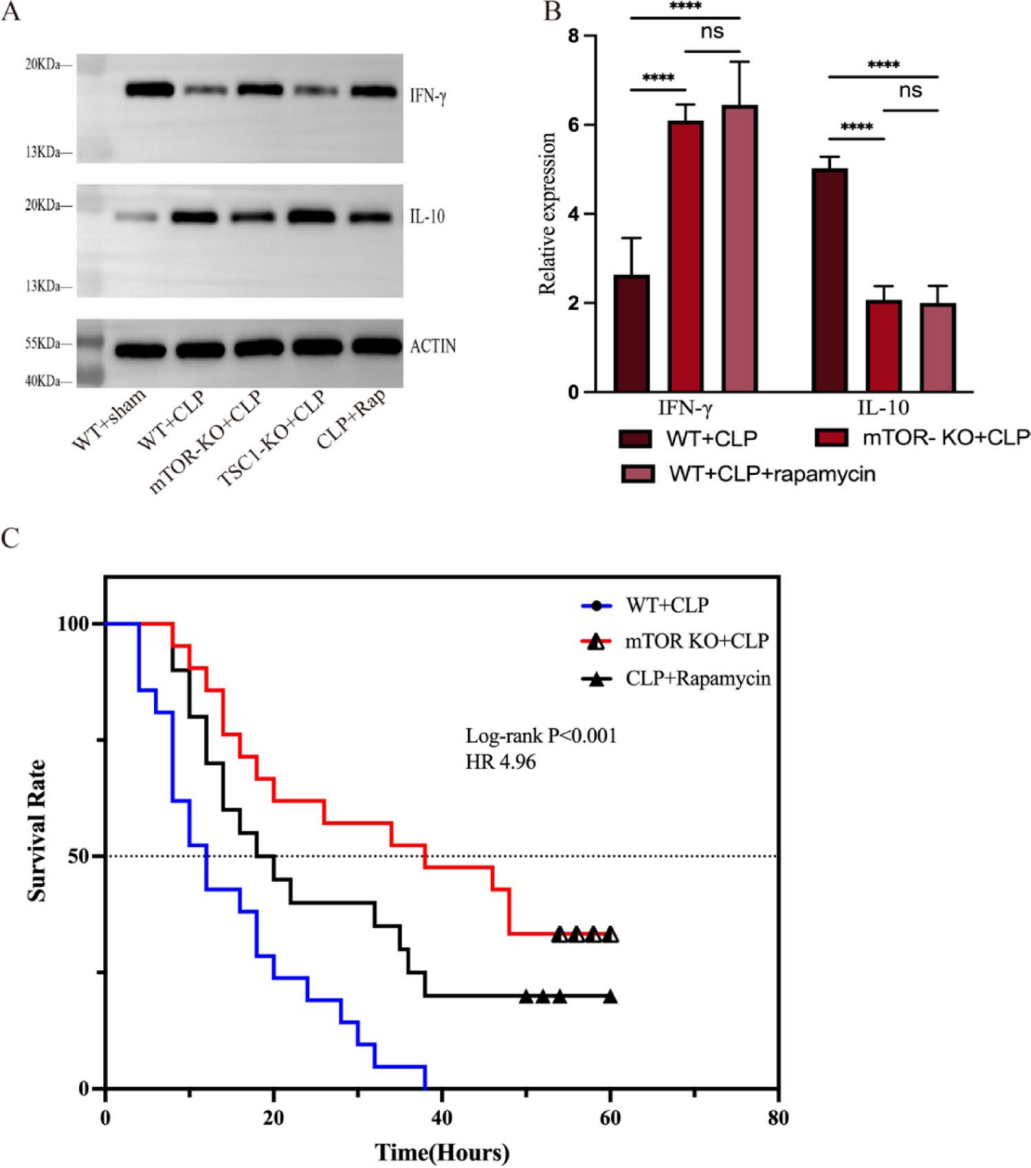
The effect of targeting mTOR in septic mice. **A**–**B** The expression level of IFN-γ and IL 10, which represent the cytokine production of cell function were measured by western blotting. **C** The state of WT + CLP, mTOR-KO + CLP, and CLP + rapamycin mice were observed and recorded every 2 h after CLP surgery until 60 h (n = 20). Mantel-Cox tests were performed using GraphPad Prism 9, exact *P* values are provided as follow: WT + CLP VS mTOR-KO + CLP: *P* < 0.0001, WT + CLP VS CLP + rapamycin: *P* = 0.012; mTOR-KO + CLP VS CLP + rapamycin: *P* = 1.64. It was deemed statistically significant when *P* < 0.05. **P < 0.01, ***P < 0.001, ****P < 0.0001

To further investigate the potential of mTOR as a clinical target, we utilized rapamycin, a specific mTOR inhibitor, to replicate and validate the outcomes initially achieved through genetic mean. Compared with WT + CLP group, the rapamycin-treated WT + CLP mice exhibited a considerable decrease the rate of CD4 + T cells ERS-apoptosis (Fig. 4G–I), which is in line with the protective impact of mTOR knockout. Explore of the functional states of cell function in which mTOR was intervened proved that targeting mTOR could revised the impaired cytokine production (indicators: INF-γ and IL-10), which we initially observed in septic mice (Fig. 5A, B). As we have proved the potential effect of mTOR on sepsis, evaluating the prognostic role of targeting mTOR is necessary. The survival time of mice among three groups: WT + CLP, mTOR KO + CLP, and CLP + rapamycin was recorded and compare at two-hour intervals. The result showed that both gene knockout and pharmacologic intervention against mTOR can improve prognosis, but the effects of knockout are more definitive (Fig. 5C).

## Discussion

Given the critical role of CD4 + T cells in the immune response, their number and function during sepsis regression are of growing concern [22]. Cell death such as apoptosis and autophagy, as well as oxidative stress, can play significant roles in the state of CD4 + T cells. And these signaling pathways are intricately interconnected. In the present study, using a cecal ligation and puncture (CLP) model, we focused on how mTOR deletion alleviates ERS-apoptosis by restoring autophagic flux provided a promising mechanism that mTOR-autophagy-ERS-apoptosis axis in CD4 + T cells during sepsis (Fig. 5). With both genetic (mTOR knockout) and pharmacological (rapamycin) approaches, we further strengthen the validity of the clinical use of targeting mTOR. By targeting mTOR, we may find out a promising way to save CD4 + T cells in sepsis to improving the prognosis of sepsis.

The endoplasmic reticulum (ER) is a site of protein processing, modification, and folding, once cellular homeostasis is disturbed, the endoplasmic reticulum stress (ERS) is initiated [23]. It has been proved that ERS can activate a range of pathways that regulate adaptive responses to environmental stresses, such as autophagy, apoptosis [24, 25]. Moreover, the potential value of ERS-apoptosis is already in the spotlight [26, 27]. In sepsis, Qi AL, Wu Y et al. found that recombinant human ulinastatin could ameliorate dendritic cell dysfunction in sepsis by interfering with ERS-apoptosis [24]. SIRT1 can similarly exert a protective effect in sepsis via the ERS-apoptosis pathway [28]. Here, we found that there is an increased proportion of apoptotic CD4 + T cells in sepsis and, as marked by CHOP and GPR, we found an increased occurrence of ERS. With 4-PBA, an ERS blocker, we further demonstrated that apoptosis of CD4 + T cells is indeed associated with ERS. Therefore, ERS-apoptosis may affect the number and function of CD4 + T cells in sepsis. We therefore intend to further explore its associated regulatory pathways. It is well known that mTOR plays a key regulatory role in cell physiology, such as endoplasmic reticulum stress, apoptosis, autophagy and other pathways [11, 29]. Further studies have proved that mTOR can regulate ERS-associated apoptosis [12]. Likewise, our previous studies have demonstrated that mTOR has a regulatory effect on ERS-apoptosis of CD4 + T cells [13]. In this study, we can see that both mTOR KO and rapamycin could reduce both ERS-related markers such as CHOP and GRP and those related to apoptosis. Taken together, the regulatory role of mTOR on ERS-apoptosis in sepsis is unquestionable.

The complex inter-regulation mechanism between autophagy and apoptosis has been attracting the attention of researchers [30]. As suggested by Shruti Singla et al., autophagy plays a protective role in a mouse model of chromosome mosaicism by regulating apoptosis [31]. Dan-Dan Zong et al. showed that autophagy activation diminishes CSE-induced apoptosis in endothelial cells, while blunting autophagy promotes apoptosis in HUVECs, and this effect is dependent on the Notch1-dependent manner [32]. In our study, the regulatory role of autophagy on ERS-apoptosis was also demonstrated with the application of bafilomycinA1, a classical inhibitor of autophagy flux. It has been demonstrated that rapamycin, an mTOR inhibitor, can enhance the apoptosis of human osteosarcoma MG-63 cells by promoting autophagy, providing a potential therapeutic direction [33]. However, in our experiments, we found that rapamycin induced autophagy, reduced the occurrence of CD4 + T cells apoptosis. The above discrepancies may be due to the different experimental models and the altered pathophysiology of the subjects under various experimental conditions. Ultimately, we conclude that mTOR can regulate the onset of ERS-apoptosis through autophagy, but the exact molecular mechanisms involved remain to be investigated.

It has been proved in our previous study that autophagosome-lysosome fusion is an important target for mTOR to regulate autophagy and thus ameliorate CD4 + T cells apoptosis in sepsis [14]. Autophagosome-lysosome fusion produces autolysosomes, which are sites for degradation of cargo when autophagy occurs [34]. Impaired autophagic flux plays a role in the pathophysiology of several situation, such as pancreatic β-cells, human parainfluenza virus type 3 (HPIV3) production [35, 36]. With bafilomycinA1 we suggested that mTOR can impact the autophagosome-lysosome fusion, and in turn affects the occurrence of autophagy, which plays an important role in CD4 + T cells apoptosis. Therefore, it is presumed that mTOR has a potential to regulate ERS-apoptosis through autophagy flux. To evidence our hypothesis, it was discovered that mTOR regulated ERS-mediated apoptosis through autophagy pathway in septic mice.

Together along with our previous articles, we have proved that mTOR affects T-cell function in sepsis by modulating pathways such as CTLA4, autophagy, and pyroptosis [37, 38]. In general, the current study suggests a new regulatory mechanism for CD4 + T cells ERS-apoptosis in sepsis, especially confirmed autophagy flux as a crucial mechanism in mitigating ER stress-induced apoptosis. We have enriched the molecular mechanisms by which mTOR regulates sepsis immune from different perspectives. We have to recognize that there are some limitations to our study. Static LC3-II and p62 levels were measured at a single time point, so we cannot distinguish increased autophagosome formation from blocked degradation. Future studies should employ Bafilomycin A1 treatment under baseline conditions or mRFP-GFP-LC3 tandem reporters to definitively clarify how mTOR deletion affects autophagic flux. Although we focused on the spleen as a major secondary lymphoid organ, we acknowledge that systemic sepsis induces inflammatory changes in multiple tissues such as the lung, liver and kidney. Future studies with larger cohorts will be required to determine whether the inflammatory pattern observed in the spleen is mirrored in other vital tissues. In the future, our further research will be further focused on verifying the effect of mTOR on CD4 + T cells, which would contribute to identifying a new target for the treatment of sepsis.

## Supplementary Information

Below is the link to the electronic supplementary material.

Supplementary Material 1

## Data Availability

The corresponding author will provide the experimental data obtained and analyzed in this study upon request.
