# Supplementary figures and images for: mTOR pathway mediates the endoplasmic reticulum stress -apoptosis of CD4+ T cell through inhibiting autophagy flux in sepsis
Source: Inflamm Res. 2026 Feb 7;75(1):34. doi: 10.1007/s00011-025-02114-4 (PMC12882968; doi:10.1007/s00011-025-02114-4)

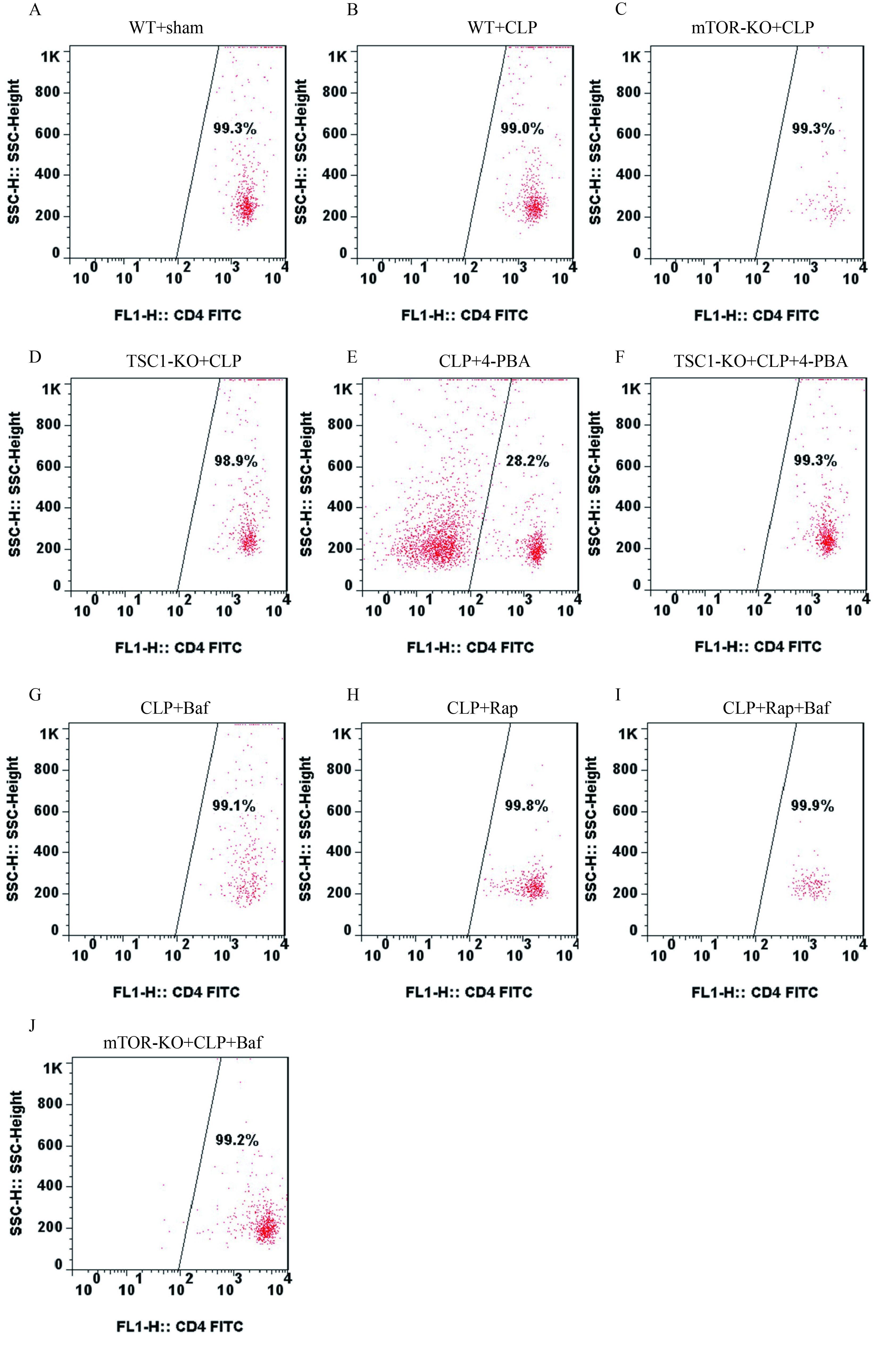

Supplement: Supplementary file 1 — Supplementary Material 1 [file 11_2025_2114_MOESM1_ESM.jpg]
